# Expressive coupling and textural dissociation in classical piano performance: evidence from the ASAP dataset

**DOI:** 10.3389/fpsyg.2026.1882570

**Published:** 2026-07-30

**Authors:** Mengjiao Yan

**Affiliations:** College of Arts and Media, Tongji University, Shanghai, China

**Keywords:** ASAP dataset, dynamics, empirical musicology, expressive timing, MIDI analysis, performance science, piano performance, rubato

## Abstract

Performance science needs empirical methods that can connect musical interpretation with measurable behavior while preserving the musical meaning of skilled performance. This study examines expressive coupling between timing flexibility and dynamic shaping, together with textural dissociation from surface note density, in public classical piano performance using the Aligned Scores and Performances dataset. The analysis extracts beat interval variability, local tempo change, MIDI velocity dynamic range, and note density from aligned annotations and MIDI note events. Composer labels are then organized into broad repertoire groupings so that timing and dynamic behavior can be compared across repertoires. Results show clear differences among these groupings, with more flexible timing and wider dynamic shaping in Romantic and late Romantic repertoires than in Baroque repertoire. The central result is an expressive coupling between timing and dynamics: timing variability is closely associated with dynamic range. At the same time, global timing flexibility is dissociated from surface note density, because note density does not account for this timing behavior. A compact feature-based prediction procedure also performs reliably above a simple frequency baseline. These findings identify expressive coupling and textural dissociation as measurable features of pianistic interpretation, showing how temporal and dynamic control operate together while remaining distinguishable from surface note density.

## 1 Introduction

Piano performance is a form of skilled action in which notation is transformed into timed, voiced, and dynamically shaped sound.

Within performance science, this transformation can be understood as the point at which musical interpretation becomes observable as organized performance behavior. Psychological accounts of music performance have long treated this transformation as more than mechanical reproduction, because expert performance depends on attention, memory, motor planning, auditory imagery, and expressive decision making (Gabrielsson, 2003). Expressive performance also carries communicative intent, so timing and dynamics become part of the performer’s interpretation rather than incidental deviations from the score (Juslin, 2003). For pianists, the task is especially demanding because polyphonic texture, touch, pedaling, and phrase direction must be coordinated within a continuous motor act. This makes piano performance a particularly suitable domain for examining how expressive control is distributed across timing, dynamics, and texture.

Research on expert musical performance has shown that memory and structure guide performance under public pressure (Chaffin and Imreh, 2002). More recent work on memory recovery has also emphasized the role of musical structure and performance cues in maintaining continuity during memorized performance (Chaffin et al., 2021). These findings are relevant to the present study because expressive timing and dynamics are behavioral traces of organized interpretive control. They record how performers distribute emphasis, tension, and release through the sounding performance. The present article develops this idea further by asking whether timing flexibility and dynamic shaping form an expressive coupling, and whether this coupling can be distinguished from the surface density of the musical texture.

Timing and dynamics are central to this behavioral record. Tactile and motor feedback shape timing accuracy in piano performance (Goebl and Palmer, 2008), and timing can be coordinated with body movement during ensemble performance (Goebl and Palmer, 2009). Expressive piano performance can also elicit physiological responses in listeners and performers (Nakahara et al., 2010). Studies of melodic expectation and perceived tension further suggest that timing is tied to musical structure and perceived affect (Gingras et al., 2016). These strands of research motivate an analysis that treats timing and dynamic control as interdependent aspects of pianistic expression, while also asking whether this interdependence can be separated from the surface density of the musical texture.

Computational work provides the tools needed for such an analysis. Earlier reviews described expressive performance modeling as a field concerned with learning systematic relations between score structure and performed timing, dynamics, and articulation (Widmer and Goebl, 2004). Later work broadened this view by comparing rule based, statistical, and machine learning approaches to expressive performance (Cancino-Chacón et al., 2018). Neural models have also been used to learn expressive musical performance from large symbolic corpora (Oore et al., 2018), and recent work continues to develop deep learning methods for expressive music modeling (Zhang, 2025). These approaches show that expressive behavior can be represented computationally, but interpretable descriptors remain useful when the research aim is to connect quantitative results with performance science and to distinguish expressive control from adjacent surface features such as note density.

Public performance datasets have made this type of empirical analysis more practical. The MAESTRO dataset supported large scale symbolic piano modeling from paired audio and MIDI recordings (Hawthorne et al., 2019). GiantMIDI Piano expanded public access to classical piano MIDI data (Kong et al., 2022). Other recent datasets have emphasized multitrack concerto performance (Özer et al., 2023), jazz piano annotations (Edwards et al., 2023), and coherent multi version analyses of Beethoven sonatas (Zeitler et al., 2024). The Aligned Scores and Performances dataset is especially relevant here because it provides aligned scores, MIDI performances, and beat annotations for classical piano repertoire (Foscarin et al., 2020). This structure allows beat timing, MIDI velocity, and note density to be studied together.

The present study focuses on observable performance behavior rather than self-report. This focus does not replace psychological constructs such as performance anxiety, self-efficacy, or perceived control. It complements that literature by measuring the timing and dynamic outcomes through which such constructs may become audible. Recent studies have refined measurement of music performance anxiety (Kenny, 2023), examined self-efficacy in music performance (Zelenak, 2024), and reviewed anxiety patterns among undergraduate musicians (Barros et al., 2022). Related validation studies have extended music performance anxiety measurement across languages and contexts (Antonini Philippe et al., 2022). Scale development and validation work has also continued in Portuguese research contexts (Barros et al., 2024). Work on feedback, perceived quality, and impostor responses further shows that performance psychology remains closely tied to public evaluation (Molińska and Rajchert, 2024). Graduate level evidence points in the same direction (Sims and Ryan, 2023). The present analysis therefore asks a narrower question about the performed signal itself, focusing on how expressive timing and dynamic shaping are organized in relation to surface note density.

The theoretical contribution is to model expressive piano behavior as an expressive coupling between beat timing, local tempo change, and dynamic range, while treating note density as a surface textural variable rather than a primary explanation of timing flexibility. This formulation separates expressive control from surface texture density at the level of measurable performance and identifies textural dissociation as a feature of pianistic interpretation. The methodological contribution is a compact and reproducible procedure that extracts beat-level timing descriptors and velocity descriptors from aligned annotations and MIDI events, then evaluates their discriminative structure with a transparent feature-based prediction model.

The rest of this paper is organized as follows. The next section describes the corpus, feature definitions, repertoire coding, statistical analysis, and prediction procedure. The results section presents corpus coverage, repertoire group differences, expressive coupling between timing and dynamics, textural dissociation from note density, and classification performance. The discussion interprets the findings in relation to expressive control, performance psychology, and computational music analysis before the conclusion summarizes the main argument.

## 2 Materials and methods

### 2.1 Dataset

The study analyzed the public Aligned Scores and Performances dataset. The corpus contains classical piano scores and performances with MIDI files, aligned score material, and beat or downbeat annotations (Foscarin et al., 2020). The analysis used MIDI performances, performance annotation files, and the metadata table to derive beat timing, MIDI velocity, and note density descriptors within the same corpus. Audio recordings were not used, because the present study focuses on symbolic performance behavior rather than acoustic signal analysis.

**Figure 1:**
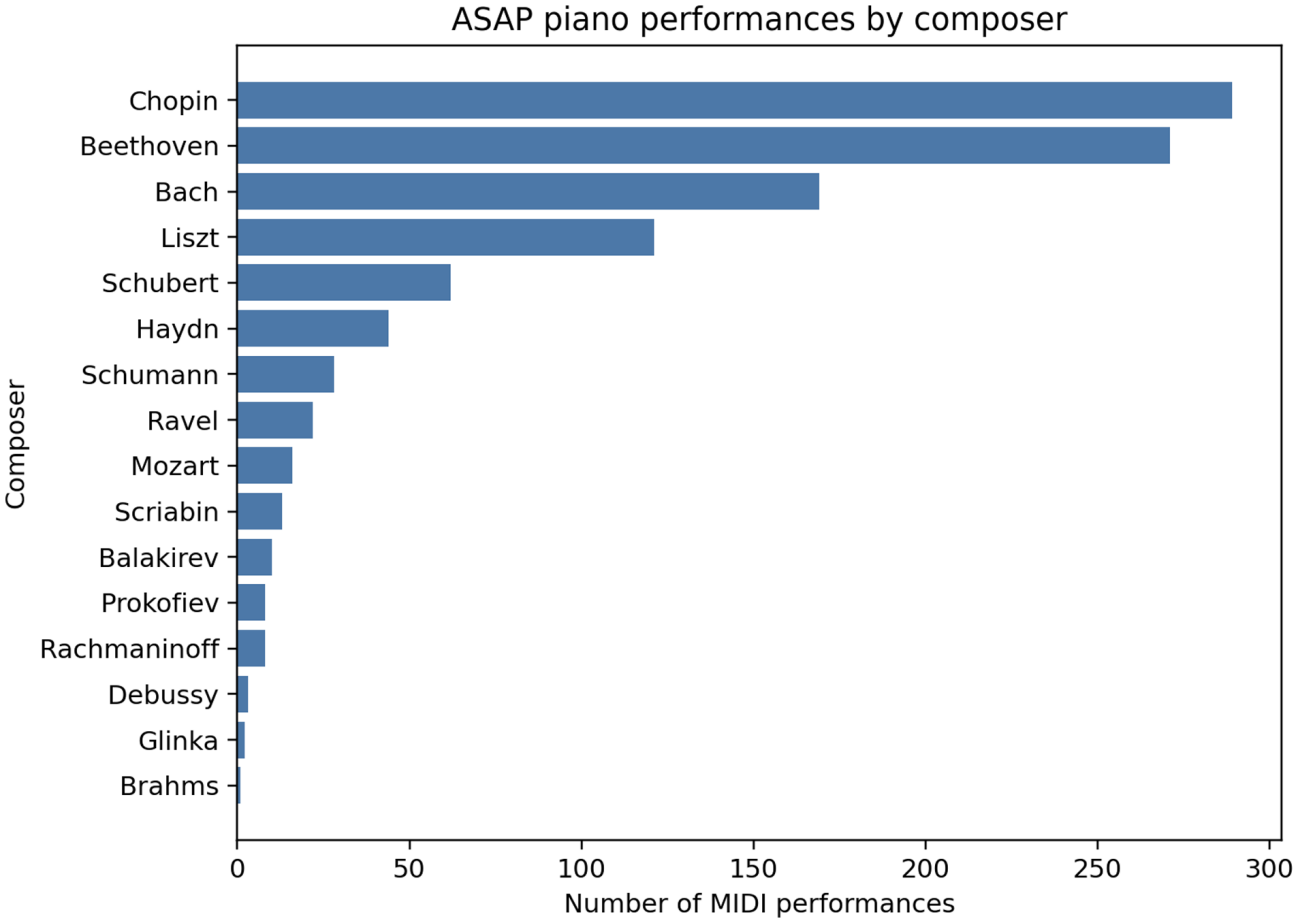
Number of analyzed MIDI piano performances by composer. The corpus is dominated by Chopin, Beethoven, Bach, and Liszt performances.

The metadata contained 1,067 MIDI performances representing 222 unique work titles. All 1,067 performances were successfully parsed and included in the analysis. The largest composer categories were Chopin with 289 performances, Beethoven with 271, Bach with 169, Liszt with 121, and Schubert with 62. Figure 1 shows the composer distribution.

### 2.2 Repertoire coding

Performances were grouped into composer based repertoire categories for descriptive and inferential comparison. Bach was coded as Baroque. Haydn and Mozart were coded as Classical. Beethoven was treated separately as Classical/Romantic because the corpus contains a large Beethoven subset and because Beethoven performance traditions often stand between Classical and Romantic idioms. Chopin, Liszt, Schubert, Schumann, Balakirev, Brahms, Glinka, and Rachmaninoff were coded as Romantic. Scriabin was coded as Late Romantic. Debussy, Ravel, and Prokofiev were coded as Modern/Impressionist. The coding scheme follows composer labels because the available metadata is organized at this level, and the resulting categories are used as broad repertoire groupings rather than exhaustive historical style classifications.

### 2.3 Feature extraction

For each performance, annotated beat times were extracted from the performance annotation file to provide the temporal basis for the timing descriptors. Let B = (b1,..., bm) denote the increasing sequence of beat times after duplicate times were removed. Beat intervals were defined as It = bt + 1 − bt. Intervals shorter than 0.05 s or longer than 10 s were removed before computing timing descriptors.

Global timing flexibility was quantified by the coefficient of variation of beat intervals, as defined in Equation 1.


$$\text{BeatCV}=\mathrm{sI}/\bar{I},$$ (1)

where Ī is the sample mean of valid beat intervals and s_I_ is their sample standard deviation. This ratio measures relative spread in beat timing and is invariant to uniform tempo scaling.

Local tempo change was calculated using Equation 2.


LocalChange=mediant∣It+1−It∣/mediantIt. (2)

This index captures successive beat to beat adjustment rather than the full spread of tempo across the performance.

MIDI note events were parsed to obtain note onset times, offset times, pitches, and velocities. Dynamic range was used as a MIDI-based descriptor of dynamic shaping and was defined in Equation 3.


DynamicRange=Q0.95(V)−Q0.05(V), (3)

where *V* is the set of note velocities and Qq denotes the empirical q quantile. Note density was computed as the number of notes divided by performance duration in seconds and was treated as a surface texture descriptor. Duration was defined as the larger value between the final MIDI note offset and the annotated beat span.

### 2.4 Statistical analysis

Descriptive statistics were computed by composer and repertoire grouping. Group differences in beat interval coefficient of variation, local tempo change, dynamic range, and note density were tested with Kruskal Wallis tests because distributions were non normal and group sizes were unequal. Spearman correlations measured associations among timing variability, local tempo change, dynamic range, and note density, with particular attention to whether timing flexibility was more closely associated with dynamic shaping than with surface texture density. The numerical analysis used Python scientific computing tools (van der Walt et al., 2011). Statistical routines were drawn from SciPy (Virtanen et al., 2020), and figures were created with Matplotlib (Hunter, 2007).

### 2.5 Feature-based prediction procedure

The computational procedure evaluated whether four features from each performance, namely beat interval coefficient of variation, local tempo change, dynamic range, and note density, preserved discriminative information about the broad repertoire groupings. These features were standardized within each training split and entered into a multinomial logistic regression model with balanced class weights. A most frequent class classifier served as the baseline. Performance was evaluated with repeated stratified cross validation, yielding 250 evaluation folds. Accuracy and balanced accuracy were compared between the feature model and baseline with Wilcoxon signed rank tests. The predictive model was implemented with scikit-learn (Pedregosa et al., 2011). Related work on automatic transcription and piano performance modeling shows that MIDI based descriptors can support structured computational analysis of performance data (Benetos et al., 2019). High resolution piano transcription research also illustrates the value of detailed symbolic event information for piano analysis (Kong et al., 2021). Hierarchical neural modeling of expressive piano performance provides a complementary predictive perspective (Jeong et al., 2019). In this study, the prediction procedure was used as a feature validation step rather than as a claim that repertoire grouping or interpretation can be reduced to classification.

## 3 Results

### 3.1 Corpus coverage

The final dataset included 1,067 MIDI performances with no parse failures. The distribution was uneven across composers, reflecting the design of the public corpus and providing an important context for interpreting subsequent repertoire group comparisons as corpus-level patterns rather than balanced historical style claims. Chopin, Beethoven, Bach, and Liszt together accounted for 850 performances, or 79.7% of the analyzed corpus. Romantic repertoire accounted for 521 performances, Beethoven’s Classical/Romantic category for 271, Baroque for 169, Classical for 60, Modern/Impressionist for 33, and Late Romantic for 13. Figure 1 presents the full composer distribution.

### 3.2 Repertoire differences in timing and dynamics

**Figure 2:**
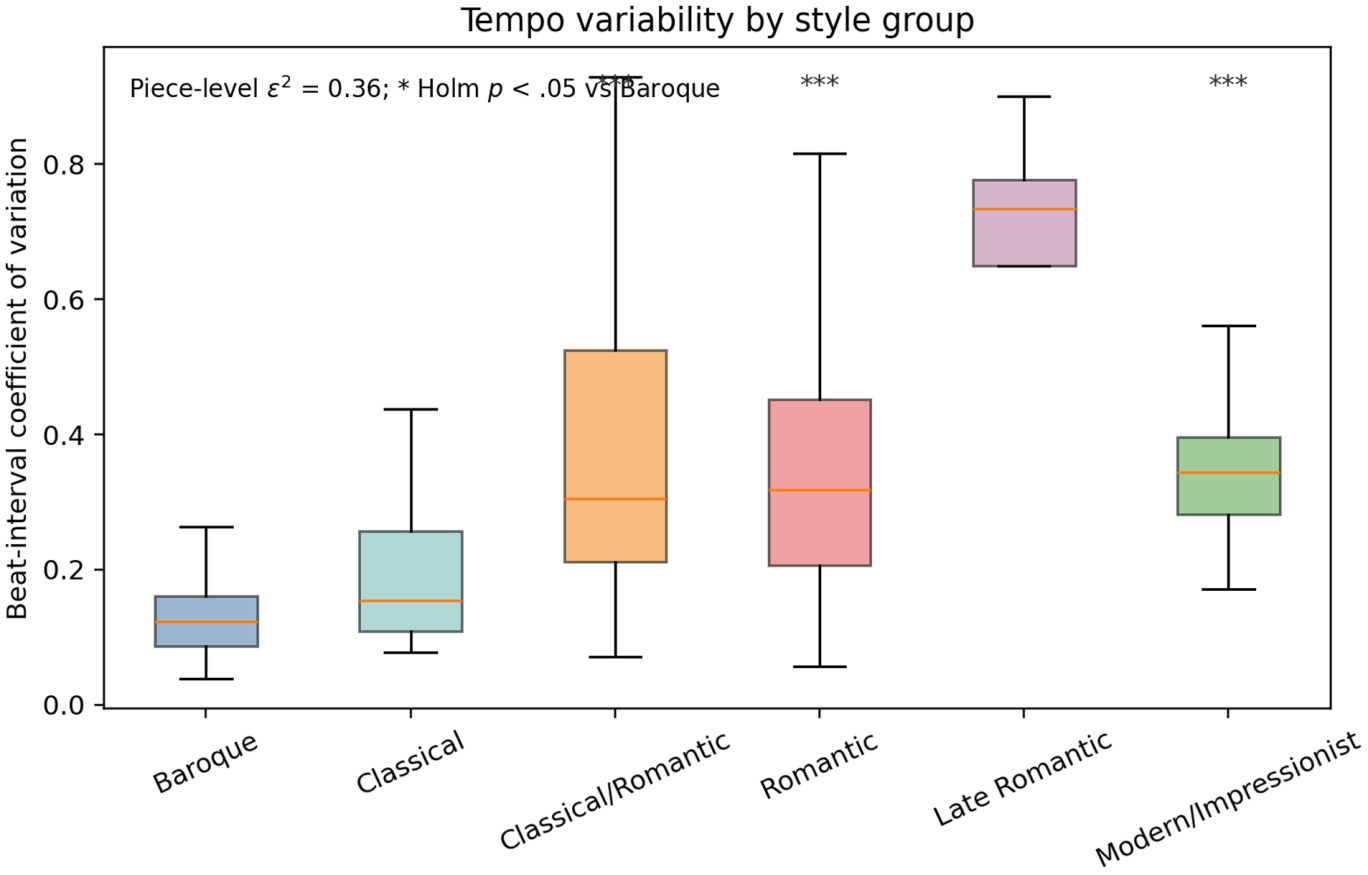
Beat interval coefficient of variation by repertoire grouping. Higher values indicate greater global timing flexibility across the performance. Outliers are omitted from the boxplot display for readability.

Tempo variability differed substantially across repertoire groupings, as shown in Figure 2. Baroque performances showed the lowest beat interval coefficient of variation with a mean of 0.133 and a standard deviation of 0.067. Classical performances were higher with a mean of 0.204 and a standard deviation of 0.133. Romantic performances showed greater tempo variability with a mean of 0.368 and a standard deviation of 0.231, while Beethoven’s Classical/Romantic category had a mean of 0.412 and a standard deviation of 0.304. Modern/Impressionist performances showed a mean of 0.336 and a standard deviation of 0.096. The Late Romantic Scriabin subset displayed the highest mean tempo variability at 0.651 with a standard deviation of 0.225. The Kruskal Wallis test confirmed significant group differences in beat interval coefficient of variation, *H* = 308.61, *p* < 0.001.

Local tempo change followed a related pattern. The mean local change index was lowest for Baroque performances at 0.033, higher for Romantic performances at 0.091, and highest for Late Romantic performances at 0.147. These results indicate that global timing flexibility and local beat to beat adjustment vary across the corpus in related but not identical ways. Group differences in local tempo change were significant, *H* = 556.77, *p* < 0.001. Table 1 summarizes the feature means by repertoire grouping, linking the timing results with dynamic range and texture density.

**Table 1:** Mean performance features by repertoire grouping.

Repertoire grouping	*n*	Beat CV	Local change	Dynamic range	Notes per sec
Romantic	521	0.368	0.091	53.84	13.13
Classical/romantic	271	0.412	0.057	54.72	9.53
Baroque	169	0.133	0.033	36.65	8.07
Classical	60	0.204	0.049	46.59	9.76
Modern/impressionist	33	0.336	0.083	59.75	12.70
Late romantic	13	0.651	0.147	64.85	10.18

Beat CV is the coefficient of variation of beat intervals. Local change is the median absolute successive interval change divided by the median beat interval. Dynamic range is the difference between the 95th and 5th percentile of MIDI velocity. Notes per sec is used as the descriptor of surface note density.

**Figure 3:**
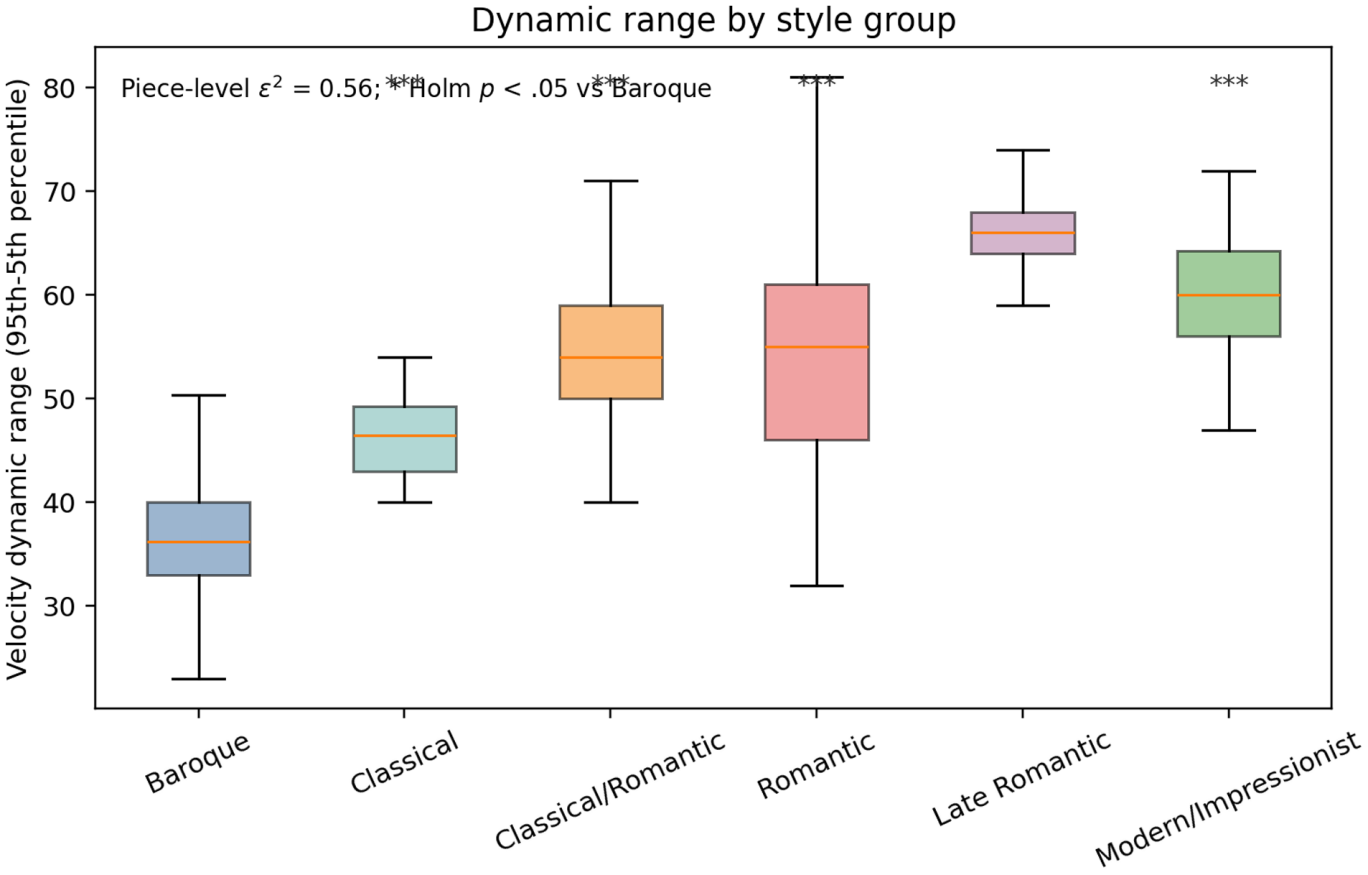
MIDI velocity dynamic range by repertoire grouping, computed as the difference between the 95th and 5th percentile of note velocities in each performance.

Dynamic range also differed across repertoire groupings, and the pattern in Figure 3 corresponded closely to the timing results. Baroque performances had the smallest mean dynamic range at 36.65 MIDI velocity units with a standard deviation of 5.92. Classical performances showed a mean of 46.59 with a standard deviation of 4.62. Romantic performances showed a larger mean dynamic range at 53.84 with a standard deviation of 9.57. Beethoven’s Classical/Romantic category was similar at 54.72 with a standard deviation of 6.53. Modern/Impressionist performances showed a mean of 59.75 with a standard deviation of 5.84, and the Late Romantic subset had the largest mean at 64.85 with a standard deviation of 5.24. The Kruskal Wallis test indicated significant group differences in dynamic range, *H* = 411.32, *p* < 0.001. Taken together, the timing and dynamic results provide the basis for examining whether coordinated expressive shaping can be distinguished from surface texture density in the following analysis.

### 3.3 Expressive coupling and textural dissociation

**Figure 4:**
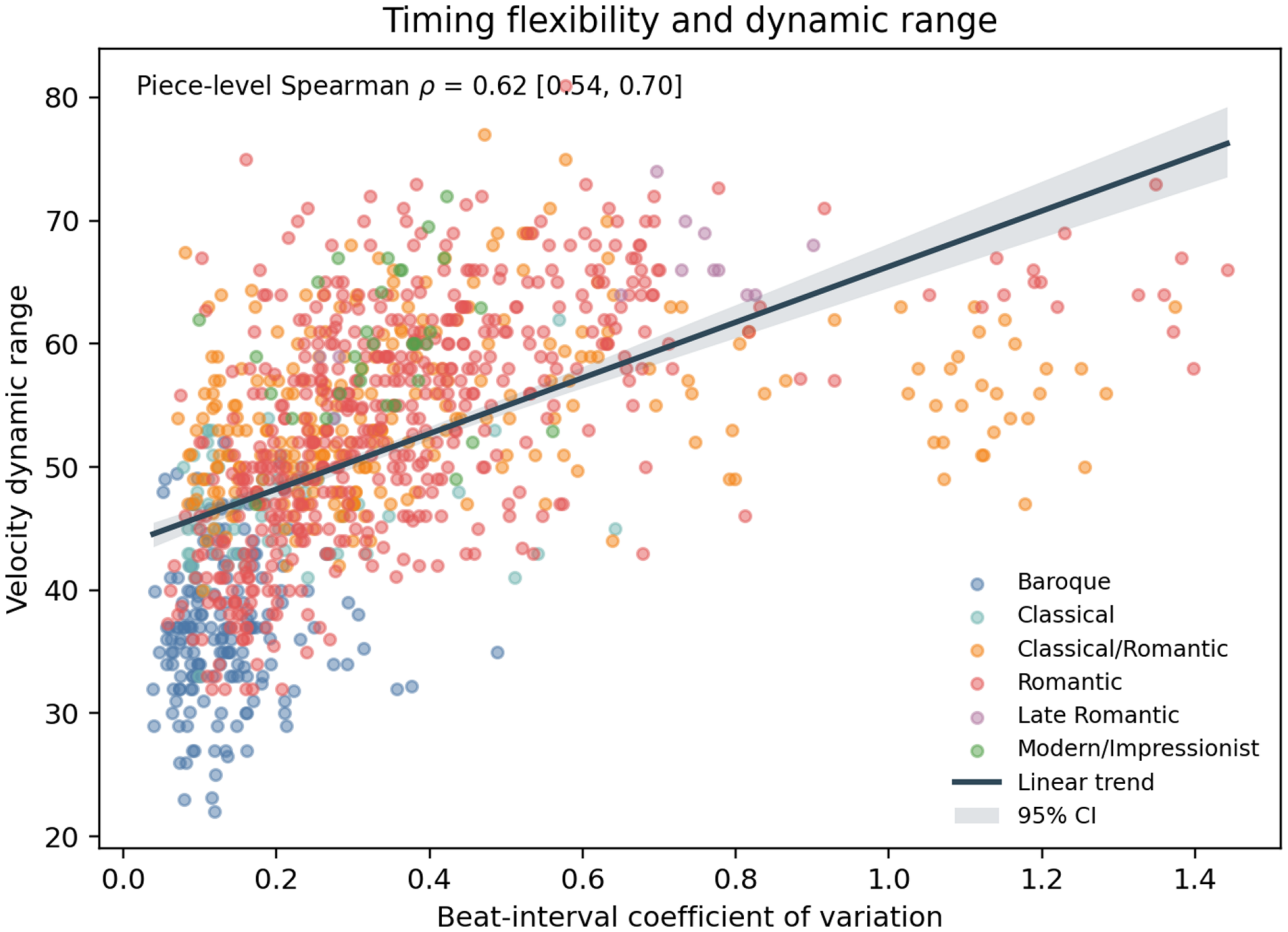
Relationship between beat interval coefficient of variation and MIDI velocity dynamic range across 1,067 piano performances. Each point represents one performance.

Across all performances, global timing flexibility was strongly associated with dynamic range. Figure 4 shows this relation at the performance level, where each point represents one MIDI performance. The Spearman correlation between beat interval coefficient of variation and dynamic range was *ρ* = 0.661, *p* < 0.001. The local tempo change index was also positively associated with dynamic range, *ρ* = 0.517, *p* < 0.001. These results indicate that performances with wider dynamic shaping tended to show greater temporal flexibility, revealing an expressive coupling between timing flexibility and dynamic range.

**Figure 5:**
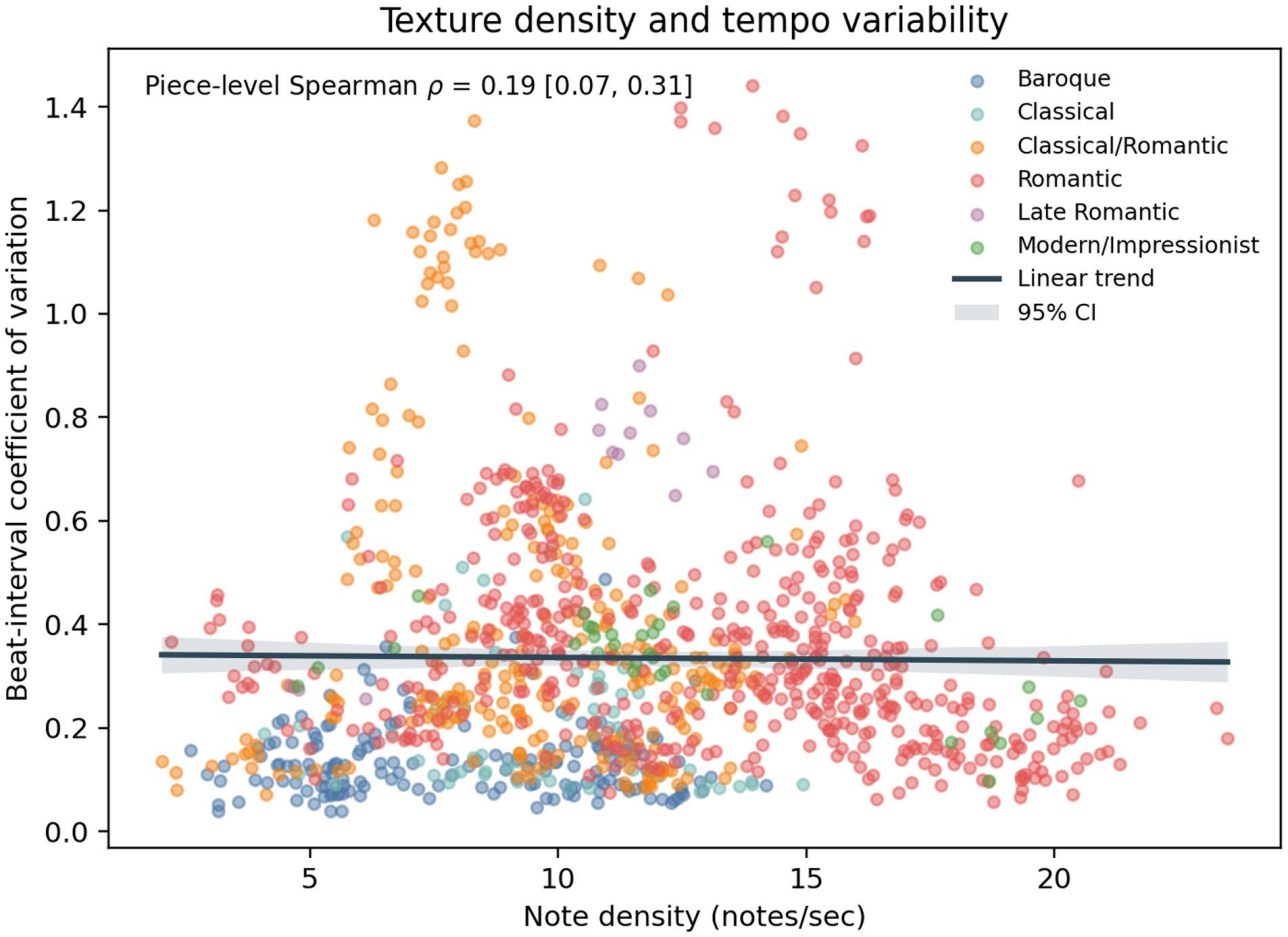
Relationship between note density and beat interval coefficient of variation. The near zero association shows that global timing flexibility is not explained by surface note density, supporting the textural dissociation identified in the corpus.

Note density behaved differently. The relation between note density and beat interval coefficient of variation was close to zero, *ρ* = 0.034, *p* = 0.271. Figure 5 makes this result visible by showing a broad spread of tempo variability across both sparse and dense textures. Note density did show a small positive association with local tempo change, *ρ* = 0.133, *p* < 0.001, but this relation was much weaker than the relation between dynamic range and timing. The density result is important because it indicates that global timing flexibility in this corpus is not simply a consequence of more notes per second. This contrast between the strong timing and dynamic association and the weak density relation forms the basis of the textural dissociation identified in this study.

### 3.4 Feature-based prediction performance

**Figure 6:**
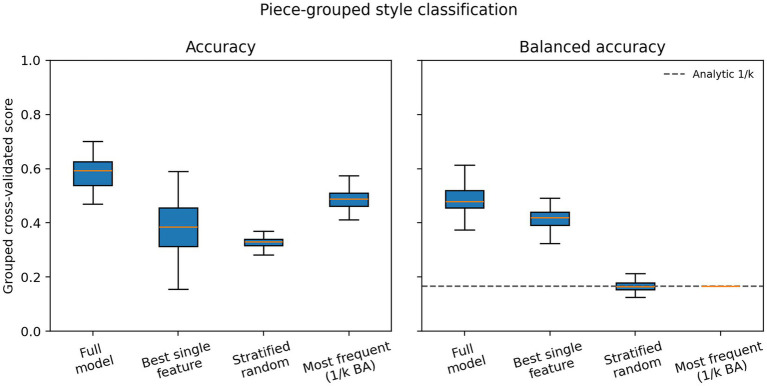
Cross-validated feature-based prediction scores for the feature model and most frequent class baseline. The separation is larger for balanced accuracy because the repertoire groupings are uneven in size.

The feature-based prediction procedure provided a validation test of whether the four extracted descriptors contain discriminative information about broad repertoire groupings. Rather than treating style as a fixed label, this analysis examined whether timing, dynamic, and density descriptors preserved meaningful corpus-level structure. As reported in Table 2, the classifier achieved a mean accuracy of 0.616 compared with 0.488 for the most frequent class baseline. The improvement was significant by Wilcoxon signed rank test, *p* < 0.001. Balanced accuracy showed a larger separation because it adjusted for the uneven class distribution. The classifier reached a mean balanced accuracy of 0.714 compared with 0.167 for the baseline, again with *p* < 0.001. Figure 6 shows the score distributions across evaluation folds.

**Table 2:** Cross-validated feature-based prediction performance.

Metric	Classifier mean	Classifier SD	Baseline mean	Baseline SD
Accuracy	0.616	0.029	0.488	0.003
Balanced accuracy	0.714	0.044	0.167	0.000

The feature model used timing, local tempo change, dynamic range, and note density to distinguish broad repertoire groupings. The baseline always predicted the most frequent class.

## 4 Discussion

The results support the central claim that expressive timing and dynamic control are coordinated in public piano performance, but they also sharpen this claim by showing a dissociation between expressive coupling and surface texture density. Baroque performances showed lower beat-level variability and smaller dynamic range. Romantic, Modern/Impressionist, and Late Romantic performances showed broader timing flexibility and wider dynamic range. Beethoven performances occupied an intermediate position in local tempo change while showing high global timing flexibility. This pattern is musically plausible because it distinguishes broad tempo shaping from successive beat adjustment, and it also fits empirical work showing that expressive timing reflects organized musical tension and structure (Rector, 2021). Empirical approaches to performance analysis have emphasized the need to connect measured performance features with close musical interpretation (Jones, 2024), and the present results follow that direction through corpus scale measurement by identifying expressive coupling and textural dissociation as measurable features of pianistic interpretation.

The association between timing variability and dynamic range is the strongest behavioral result. It suggests that pianists shape musical motion through linked temporal and dynamic processes. Dynamic expansion and tempo flexibility can both support phrase motion, arrival, emphasis, and release. This interpretation is consistent with research that treats expression as a multidimensional performance behavior rather than a single acoustic cue (Juslin, 2003). It also aligns with computational models in which expressive performance is learned as a pattern across multiple performance dimensions (Cancino-Chacón et al., 2018). The present analysis adds a directly interpretable feature view by showing that a small set of timing and velocity descriptors already captures a strong relation between temporal and dynamic control, thereby identifying expressive coupling as a measurable component of pianistic interpretation rather than a purely descriptive metaphor.

The weak relation between note density and global timing flexibility gives the result its main interpretive force. Dense textures may demand more physical effort and perceptual planning, yet the corpus did not show a meaningful relation between density and global timing flexibility. This separates expressive timing from a simple account based on texture load. The small positive relation between note density and local tempo change may reflect local motor adjustment in denser passages, but it does not explain the broader timing range across performances. The contrast between Figures 4, 5 therefore clarifies the argument: dynamic shaping is closely tied to timing flexibility, while surface density is not.

This textural dissociation is important for performance science because it shifts the interpretation of timing variability from a broad stylistic observation to a more specific behavioral mechanism. If global timing flexibility were mainly associated with note density, rubato-like behavior could be understood largely as a response to surface texture, technical load, or motor complexity. The present pattern points elsewhere: timing flexibility is closely aligned with dynamic shaping, yet remains largely independent of the number of notes per second. This distinction moves the analysis beyond the expected observation that Romantic and Late Romantic repertoires show greater flexibility than Baroque repertoire. It separates two explanatory levels: expressive coupling, which describes the alignment between timing flexibility and dynamic range; and textural dissociation, which shows that this alignment is not reducible to surface density. For performance science, this provides behavioral evidence that expressive interpretation has an organizational logic of its own. Skilled performance is therefore not only a matter of accuracy, fluency, or technical complexity, but also of coordinating temporal and dynamic energy so that phrase direction, arrival, suspension, and release become perceptible in sound.

The feature-based prediction results add a validation test of the same structure. A transparent classifier using only four features performed reliably above the baseline, especially after class imbalance was addressed through balanced accuracy. This does not turn repertoire grouping into a fixed stylistic label, and it does not reduce interpretation to classification. It shows that the extracted descriptors preserve enough structure to distinguish broad repertoire groupings better than frequency alone. That result is consistent with the descriptive statistics and strengthens the link between the empirical pattern of expressive coupling and the computational procedure used to measure it. In this sense, the prediction analysis supports the sufficiency of the selected descriptors without making classification the central theoretical claim.

The broader performance psychology literature provides an important frame for these behavioral findings. Music performance anxiety research has developed detailed measurement instruments (Kenny, 2023), cross cultural validations (Dias et al., 2022), and accounts of psychological flexibility and perceived quality (Viator et al., 2024). Work on public performance simulation has also created richer environments for studying performance pressure (Waddell et al., 2025). The present study does not infer inner states from MIDI data. It instead provides an observable behavioral layer that can be connected to those psychological constructs in future empirical designs. Timing and dynamics are observable outcomes, and their coordination offers a useful bridge between computational corpus analysis and performance science. By distinguishing expressive coupling from surface note density, the study offers a more precise account of how interpretive control becomes audible in skilled piano performance.

Several limitations should be noted. First, the repertoire categories used in this study are based on composer labels and should be understood as broad corpus-level groupings rather than exhaustive historical style classifications. Second, MIDI velocity provides a useful symbolic descriptor of dynamic shaping, but it should not be equated directly with acoustic loudness or perceived intensity. Third, the analysis identifies observable timing and velocity patterns, but it does not directly reconstruct performers’ intentions or psychological states. Future research could connect the present corpus-level findings with phrase level score analysis, performer reports, acoustic recordings, and listener perception. These limitations clarify the level of the present claim: the study identifies expressive coupling and textural dissociation as measurable behavioral patterns in public piano performance.

## 5 Conclusion

This study examined expressive timing and dynamic control in a public corpus of classical piano MIDI performances. The results show that broad repertoire groupings differ in timing flexibility, local tempo change, dynamic range, and note density. They also show that global timing flexibility is closely related to dynamic shaping, while note density does not account for the same timing behavior. This pattern identifies an expressive coupling between timing and dynamics, together with a textural dissociation between global timing flexibility and surface note density. A compact prediction procedure based on these descriptors performs reliably above a simple baseline, which indicates that the features preserve meaningful performance structure. The findings support an account of pianistic expression in which performers coordinate temporal and dynamic control as part of a unified interpretive behavior. In this sense, the study provides a reproducible empirical pathway for examining how pianistic interpretation becomes observable as measurable performance behavior in a public corpus.

## Data Availability

The original contributions presented in the study are included in the article/supplementary material, further inquiries can be directed to the corresponding author.
